# What keeps health professionals working in rural district hospitals in South Africa?

**DOI:** 10.4102/phcfm.v7i1.805

**Published:** 2015-06-26

**Authors:** Louis S. Jenkins, Colette Gunst, Julia Blitz, Johan F. Coetzee

**Affiliations:** 1 Division of Family Medicine and Primary Care, Faculty of Medicine and Health Sciences, Stellenbosch University, South Africa; 2 Western Cape Department of Health, South Africa

## Abstract

**Background:**

The theme of the 2014 Southern African Rural Health Conference was ‘Building resilience in facing rural realities’. Retaining health professionals in South Africa is critical for sustainable health services. Only 12% of doctors and 19% of nurses have been retained in the rural areas. The aim of the workshop was to understand from health practitioners why they continued working in their rural settings.

**Conference workshop:**

The workshop consisted of 29 doctors, managers, academic family physicians, nurses and clinical associates from Southern Africa, with work experience from three weeks to 13 years, often in deep rural districts. Using the nominal group technique, the following question was explored, ‘What is it that keeps you going to work every day?’ Participants reflected on their work situation and listed and rated the important reasons for continuing to work.

**Results:**

Five main themes emerged. A shared purpose, emanating from a deep sense of meaning, was the strongest reason for staying and working in a rural setting. Working in a team was second most important, with teamwork being related to attitudes and relationships, support from visiting specialists and opportunities to implement individual clinical skills. A culture of support was third, followed by opportunities for growth and continuing professional development, including teaching by outreaching specialists. The fifth theme was a healthy work-life balance.

**Conclusion:**

Health practitioners continue to work in rural settings for often deeper reasons relating to a sense of meaning, being part of a team that closely relate to each other and feeling supported.

## Abstract (fr)

**Qu'est-ce qui encourage les professionnels de la santé à travailler dans les hôpitaux de districts ruraux en Afrique du Sud?**

**Contexte:**

Le thème de la Conférence sur la Santé rurale en Afrique australe en 2014 était “Développer la Résilience pour faire face aux réalités rurales’. Il est crucial de retenir les professionnels de la santé en Afrique du Sud pour avoir des services de santé durables. Seuls 12% des médecins et 19% des infirmiers ont été retenus dans les zones rurales. Le but des ateliers était de demander aux professionnels de santé pourquoi ils continuaient à travailler dans les zones rurales.

**Atelier-conférence:**

L'atelier comprenait 29 médecins, gestionnaires, médecins de famille académiques, infirmières et collaborateurs cliniques d'Afrique du Sud, avec une expérience de travail de trois semaines à 13 ans, souvent dans des zones rurales reculées. Au moyen de la technique du groupe nominal, la question suivante a été examinée, ‘Qu'est-ce qui vous fait aller au travail chaque jour? ‘. Les participants ont réfléchi sur leur situation de travail et ont fait une liste et évalué les raisons importantes pour continuer de travailler.

**Résultats:**

Cinq thèmes principaux sont apparus. La raison la plus forte pour rester travailler à la campagne était un objectif commun, provenant d'un sens profond. La seconde était de travailler en équipe, où le travail d’équipe était lié aux attitudes et relations, au soutien des spécialistes itinérants et à la possibilité de mettre en œuvre les compétences cliniques individuelles. La troisième était une culture de soutien, suivie par des opportunités de croissance et de développement professionnel continu, telles que la formation par des spécialistes de sensibilisation. Le troisième thème était un bon équilibre entre la vie familiale et professionnelle.

**Conclusion:**

Les professionnels de santé continuent à travailler dans les zones rurales pour des raisons souvent plus profondes liées à leur raison d’être, leur appartenance à une équipe dont les membres sont étroitement liés et au sentiment d’être soutenus.

## Introduction

### Background

The theme of the Southern African Rural Health Conference held at the Worcester Rural Clinical School in South Africa in September 2014 was ‘Building resilience in facing rural realities’. It was co-hosted by the Rural Doctors’ Association of Southern Africa (RUDASA), Rural Rehab of Southern Africa (RURESA), and the Professional Association of Clinical Associates in South Africa (PACASA). Retaining health professionals in the district hospitals in the 52 health districts in South Africa is critical for sustainable health services.^1^ South Africa joins many countries in Africa and other parts of the world where the number of doctors is below 1/1000 population, with the ‘ratio of physicians per 1000 population essentially unchanged between 2004 (0.77) and 2011 (0.76)’.^2^ Whilst the number of graduating doctors in South Africa increased by 18% between 2000 and 2012, with many doctors leaving the country, the absolute numbers have not increased. In addition there has been a shift in gender parity to more women; and in racial parity to ‘more black Africans and persons of mixed ancestry, and fewer whites and Indians’.^2^

Various strategies for recruitment and especially retention of health staff have been described, but the question was what local health professionals in South Africa thought and believed about retaining staff in a district hospital: what keeps them working there, despite the many challenges. In other words: What pull factors override all the push factors of working in low-resourced rural environments? Some of the challenges/push factors include: the quadruple burden of disease found in the HIV and tuberculosis epidemic; a high rate of violence and road accidents; high maternal and child mortality; and a large non-communicable disease epidemic resulting from a Western lifestyle.^3^ Other challenges include: an absolute shortage of doctors and other health professions; high turnover of junior doctors; and inequity between urban and rural, private and public practice, with only 12% of doctors and 19% of nurses being retained in the rural areas and an overall 30% of doctors caring for 85% of the uninsured population in the country.^4^ The necessity of ensuring good leadership and health management to address issues like supply shortages, corruption, accommodation and infrastructure, as well as continuing medical education and ‘career pathing’ opportunities for health professionals, has been recognised as a compounding factor in retention.^5^ South Africa has the highest Gini coefficient (0.7) in the world, with poverty, unemployment and poor education in communities often providing the background in which health challenges are embedded.^2^

Strategies to retain health professionals have been developed internationally by the World Health Organization, Australia, Canada, and South Africa.^6,7,8^ Examples of the strategies employed in South Africa include: the introduction of a compulsory community service year for doctors and allied health professionals and, more recently, also for nurses; the initiation of the midlevel clinical associate programme; the start of the National Health Insurance project; and the upgrading of health infrastructure in the country. Non-government organisations, for example African Health placement (AHP) and local academic institutions, for example, the Rural Health Advocacy Project of the University of Witwatersrand, have also played a role.^9,10^ However, these changes do not translate to improved retention of staff overnight and many of the challenges are still a painful daily reality for health workers at the coalface. So the question remains, why do doctors, allied healthcare workers and nurses remain in rural areas in South Africa?

### Conference workshop

A workshop was conducted and consisted of 29 doctors, managers, academic family physicians, nurses and clinical associates from all over South Africa, with work experience ranging from three weeks to 13 years, often in deep rural districts. Using the nominal group technique,^11^ the following question was explored, ‘What is it that keeps you going to work every day?’ Participants reflected on their own work situation, then listed and rated the most important reasons for continuing to work in the challenging health system as mentioned above. The focus was on ‘retention in work’ rather than what drives healthcare workers to apply for work in a rural area. After clarification and prioritisation of emerging themes in small groups, all participants reached consensus on the five most important reasons for continuing to work in a small rural district hospital.

## Results

The five main themes that emerged are now discussed in more detail.

### Shared purpose

A shared purpose, emanating from a deep sense of meaning, was the strongest reason for staying and working in a rural setting. ‘I am making a difference’ and ‘I know that I am called to do this’ were typical comments. People felt that they were making, or wanted to make, a difference to the health of the local community. Some spoke about ‘going back home’, having been recruited from that rural area. These health practitioners had a very particular identity within their communities of practice which were being formed all the time whilst immersed in the rural realities, a process well described by Wenger.^12^

### Working in a team

Working in a team featured as second most important. It was more than just having a team, but also related to attitudes and relationships, the wider team of support coming from the visiting specialists, support to implement clinical skills within that team and even how the team fits into one's personal and community life. Visiting specialists are general specialist doctors in the various clinical disciplines who are usually based in the regional referral hospital and who reach out to the surrounding district hospitals in the periphery on a regular basis, to provide clinical service and teaching to the medical officers and family physicians. One recently-qualified family physician emphasised the fact that ‘they [*visiting general specialists*] knew me’ as being one of the keys to successful completion of her family medicine registrar training in a rural area. ‘Teamness’ involves much more than a general team spirit, but relates to a transformation in people's minds around understanding team members’ passion, using the ‘we’ language and celebrating and rewarding people and events for mutual benefits.^13,14^ Being part of a team allows a person the space to celebrate successes and to debrief from stressful encounters. It relates to becoming part of the community that the health practitioner envisioned initially and attracted him or her toward the actual community – a process that Wenger described as a healthy tension between reification (translating the abstract concept of teamwork in the brain into a visible reality) and participation (being a team player) playing itself out in everyday rural living and working.^12^ This sense of belonging, or cohesion, being the ‘social glue’ that binds people together, with opportunities to develop, not just professionally, but also personally, came out strongly.

### Culture of support

A culture of support, closely related to ‘teamness’, was the third dominant theme. Interpersonal work relationships, such as ‘sometimes we just share our frustrations or joys around a cup of coffee’, were highly valued. Receiving feedback from managers, colleagues and patients were specifically mentioned as one powerful means of support. Giving feedback does not have a financial cost, but it does imply a personal time commitment, care about team members and a leadership style that embraces vulnerability toward colleagues. Giving honest feedback with a view to developing people and considering their self-esteem, whilst also maintaining high clinical standards, is a very specific skill that needs some training and coaching.^15^ These characteristics of prioritising social relationships and seeing the work community as an extension of one's personal community, together with a need for feedback, flexibility and structure, are all strong features of the millennial generation, which refers to people who turned 18 years of age in the year 2000, reflecting the age group of the majority of participants in the workshop.^16^

### Opportunities for growth

Professionally, with opportunities for growth (the fourth theme), people wanted a challenging and stimulating work environment, with continuing professional development, specific teaching by outreaching specialists and having the opportunity to use their clinical skills. There was a discussion in one group about the fact that working in rural areas affords one the benefit of being able to apply all the skills one has acquired during training as a doctor.

### Work-life balance

Discussions regarding a healthy work-life balance (the fifth theme) revolved around balancing a sense of structure (what needs to be done) and flexibility (what can be adapted to allow more time with family). Work does not occur outside life; people live as they work and, in rural areas, often *where* they work. So it may be more appropriate to refer to a work-family balance, or even talk about a work-family border theory, as Clark described, where people are border-crossers who make daily transitions between the two worlds of work and family, determining those boundaries, shaping their environment and being shaped *by* the environment.^17^

## Discussion

The main themes emerging from the workshop paint a picture of a healthcare practitioner with a shared purpose, working in a supportive team, having personal and professional opportunities for growth, within a healthy work-life balance. These themes reflect closely the literature on retention and should inform future planning for attracting and keeping health workers.^18^ These are also established key components of building resilience, which was the main theme of the conference. Close relationships, facilitative leadership, support from outreaching specialists, teamwork, work environment and a culture of learning have all been cited as dominant attributes of the workplace that increase retention.^18^

These themes could also be linked to the empowerment theory which Zimmerman presents as:

… both a value orientation for working in the community and a theoretical model for understanding the process and consequences of efforts to exert control and influence over decisions that affect one's life, organizational functioning, and the quality of community life.^19,20^

**FIGURE 1:**
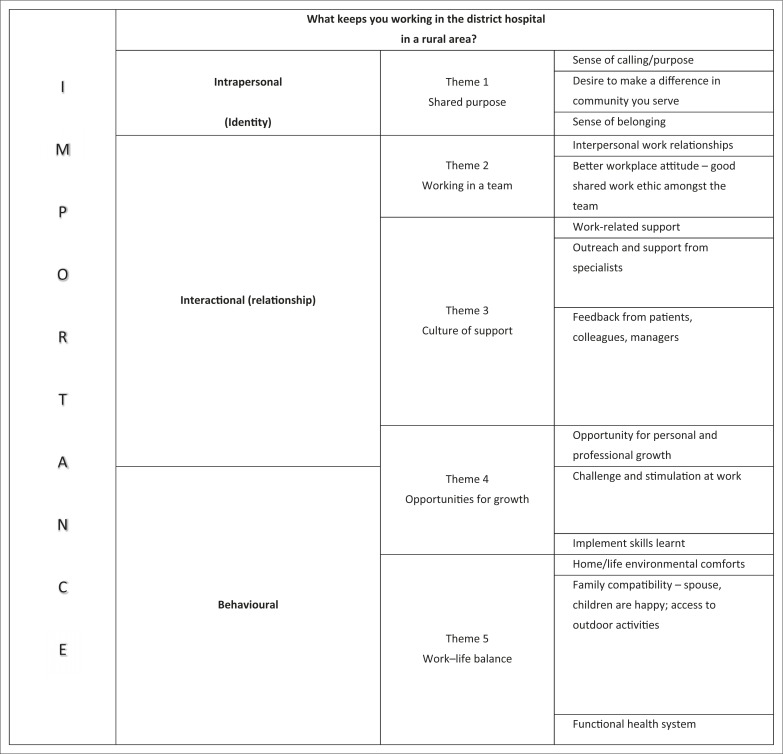
The main themes that keep health workers working in rural district hospitals, in order of most important to least important, as voted by the workshop participants

This model of psychological empowerment includes intrapersonal, interactional and behavioural components.^20^ Figure 1 illustrates how the themes can be linked with these components of the empowerment theory as a potential framework to explain why health workers stay in rural district hospitals. It may help to inform retention strategies and even be applied to similar rural contexts elsewhere, where it has been shown that it is the total personal and professional experience, not salary alone, which impacts retention.^21^

District managers and healthcare practitioners should include these themes in their discussions of retention strategies in order to create awareness and make these themes explicit, within the challenges of working in rural areas.

## Conclusion

The aim of the workshop was to determine what keeps health practitioners working in rural health districts. Five main themes were identified, which included having a sense of shared purpose, working in a team, within a culture of support, having opportunities for growth and maintaining a healthy work-life balance. These should serve to inform future retention strategies of health practitioners in rural health settings.
